# Industrial green total factor productivity in China’s Yangtze River Economic Zone: Temporal and spatial difference analysis based on 108 panel data

**DOI:** 10.1371/journal.pone.0259366

**Published:** 2022-07-01

**Authors:** Xin Zhang, Shen Zhong, Fengge Yao, Yuexin Li, Jian Li

**Affiliations:** 1 Honors School, Harbin University of Commerce, Harbin, Heilongjiang Province, China; 2 School of Finance, Harbin University of Commerce, Harbin, Heilongjiang Province, China; Institute for Advanced Sustainability Studies, GERMANY

## Abstract

As China’s strategic support belt, the green development of industry in the Yangtze River Economic Zone is of great significance to promote the construction of China’s ecological civilization, build a modern industrial system and accelerate high-quality economic development. The study of green total factor productivity of industry in the Yangtze River Economic Zone has important theoretical and practical value for exploring the green development path of China’s industry. This Paper takes the Yangtze River Economic Zone, a key strategic region in China, as the research object, selects the input and output data of industrial production from 2006 to 2018, based on DEA model. To construct an MML index considering expected and unexpected output, and to quantitatively analyze the changes of industrial GTFP in the Yangtze River Economic Zone. The results show that: (1) During the sample period, the industrial green total factor productivity in the Yangtze River Economic Zone shows the spatial characteristics of differential growth and the temporal characteristics of volatile growth. It shows the fluctuation characteristics of “N” shape. (2) According to the order of "upper, middle, and lower" reaches, the spatial pattern of industrial green total factor productivity is characterized by "lower ladder". But the difference between the upper and middle reaches is small. (3) Cities with higher green total factor productivity and lower green total factor productivity each form the characteristics of "club convergence" of spatial agglomeration. (4) Technological efficiency and technological progress efficiency have heterogeneous effects on different river basins in the upper, middle, and lower reaches, and technological progress efficiency is conducive to promoting the evolution of green total factor productivity to a high level. According to the above empirical results, this paper finally puts forward the policy recommendations to improve the industrial green total factor productivity of the Yangtze River Economic Zone and the policy recommendations to reduce the industrial differences between the Yangtze River Economic Zone.

## 1. Introduction

As an important hub of the Marine Silk Road in the 21st century, the Yangtze River Economic Zone has developed rapidly and played a strategic role in China’s social and economic development [1]. According to the China Statistical Yearbook, the industrial added value of the Yangtze River Economic Zone reached 4747.354 billion yuan in 2007 and 18234.18 billion yuan in 2019. It increased by about 2.84 times, accounting for 47.3% of China’s industrial output. However, with the rapid development of industry in the Yangtze River Economic Zone, there have been serious environmental pollution [2] and unbalanced industrial development in various river basins. Considering The relationship between industrial development and resource and environment productivity in the Yangtze River Economic Zone, as well as the specific situation of industrial development in each basin, this paper focuses on the space-time development characteristics of industrial green total factor producer in the long-term economic zone and the effect of decomposition factors. It has certain theoretical and practical significance.

At present, there are two main problems in the urban industry of the Yangtze River Economic Zone. Firstly, there is serious environmental pollution in the industrialization development of the Yangtze River Economic Zone, which seriously restricts the sustainable economic development of the Yangtze River Economic Zone and China [3,4]. The total wastewater discharge in the Yangtze River Basin accounts for more than 40% of China’s total, and the discharge intensity of ammonia nitrogen and sulfur dioxide per unit area is 1.5 to 2.0 times of China’s average level (China Sewage Treatment Network, 2018).

Secondly, there are significant differences in the development of industry in different cities. In the early stage of China’s reform and opening up, the implementation of regional development strategy not only promoted the industrial development of the eastern, but also significantly widened the gap between coastal and inland regional industries [5]. Later, with the gradual advancement of the "industrial transfer" policy and the different industrial bases of regional environmental carrying capacity, the industrial differences between cities in the upper, middle, and lower reaches of the Yangtze River Economic Zone are more obvious. Based on the above two phenomena, this paper uses the Meta-frontier Malmquist Luenberger index (MML index) with the addition of industrial "three wastes" as the negative output, and the Data Envelopment Analysis (DEA) based on the Meta-frontier production function. Considering the regional heterogeneity among groups, it calculates the change of industrial green total factor productivity (Green total factor productivity of industry in Yangtze River Economic Zone, GTFP) and its causes in different regions of China’s Yangtze River economic zone from 2006 to 2018.

The second part of this paper introduces the research of the relevant literature, the third part introduces the theoretical basis, and the fourth part is the data description, including the selection of indicators and data sources. The fifth part is empirical analysis, the conclusion and related policy recommendations in the sixth part of the description.

## 2. Literature review

The concept of total factor productivity was first put forward in the book Productivity Accounting by Shilon Davies (1954). It refers to the factors that can contribute to economic growth besides the input of all tangible factors of production (including capital, labor, land, etc.), that is, the ratio of total output to total factor input, which cannot be explained by the productivity of tangible input factors in economic growth [6]. Since then, academics have generally believed that total factor productivity is an indicator of scientific and technological progress reflecting the comprehensive level and its changes of productivity. At present, the research on industrial green efficiency mainly focuses on the following two aspects:

One is the measurement method of industrial green efficiency, which is mainly divided into two kinds: parameter method and non-parameter method. The Parameter method is mainly the stochastic frontier method (SFA) [7,8], through the preset function, the random disturbance term is introduced. Calculating growth structures using metric methods [9,10]. Honma [11] calculate the efficiency of industrial green development in Japan by using stochastic frontier analysis. Makridou [12] used random frontier analysis to measure the efficiency of industrial green production in Europe. However, the traditional SFA method has some defects [13], and it is difficult to reasonably control the endogenous problems of input-output variables and functions. It is necessary to ensure that the preset production function is reasonable [14]. The non-parametric method is represented by data envelopment analysis (DEA), which was first proposed by (Fare, 1951), and then studied and developed by Acaenes et al [15] based on the concept of relative efficiency. This method can overcome the strong hypothesis bias of model setting in the parameter method and decomposes the parameter of total factor productivity source, so it is more applied to the study of efficiency and productivity considering environmental factors [16]. It is also the main method to measure the green efficiency of industry at present, and is often used in conjunction with Malmquist-Luenberger index. Coli M et al [17] assessed the environmental efficiency of Italian provinces in 2004 by using the non-parametric efficiency measurement method in the DEA model. Fare R et al [18] uses the DEA model to calculate the environmental efficiency index based on the cross-section data of 17 OECD countries in 1990. The Environmental performance of these countries was evaluated. Jefferson [19] produced panel data for industrial enterprises above the scale, including the capital, labor, and industrial added value. Estimating the green total factor productivity of China’s industry from 1998 to 2005. Ramanathan [20] uses the Malmquist-Luenberger index to analyze the environmental performance of 17 countries in the Middle East and North America. As for pollutants, some scholars put forward that pollution emission is an unexpected output index, and Pitman [21] first tried to incorporate environmental factors into the model of productivity measurement and used the cost of pollution control as an "unexpected output" to measure the efficiency of Wisconsin paper mills in the United States. (Fare et al,2001) regards energy consumption as an input index, environmental pollution as an unexpected output, and uses directional distance function to measure total productivity with resource and environmental constraints. Mohtadi [22] constructs an evaluation from the perspective of input and output These include resource input, expected output and unexpected output. The level of economic development is expected output, but not expected output refers to environmental pollution indicators. Another part of scholars regards environmental pollution as a dependent variable. The level of green development was measured [23–25], which set pollution emissions as dependent variables with 41 developed, measured against highly polluting industries and enterprises in developing countries and 26 OECD countries as sample data.

In the empirical analysis, Shiyi C [26] uses the directional distance function to analyze the industrial total factor productivity under the environmental regulation, and the total factor productivity included in the environmental constraints is much lower than the traditional method estimate. Since then, a large number of research literature have emerged in this field. Based on Indonesia’s low-carbon economic development goals, Rusiawan [27] has studied the impact of carbon intensity on green total factor productivity GTFP. It is concluded that reducing CO2 emissions can improve green total factor productivity GTFP. Boussemart J C [28] selected 30 OECD countries and used Luenberger productivity index to consider the evolution of green productivity under carbon emissions. From a certain point of view, the green total factor productivity of the whole national economy is studied. Considering "environmental pollution" and "innovation failure", Liu C et al [29] constructs an improved SBM DEA efficiency measurement model to measure the green technology innovation efficiency of China’s high-tech industrial clusters. Zhang, Meng and Wang [30–32] constructed a variety of de models, non-radial DEA models, and improved DEA models to calculate the industrial ecological efficiency of various provinces in China. Based on various extended and improved DEA methods, (Emrouznejad and Yang, Fujii and Managi, Han et al., Yan and Fang, Shao et al. [33–37] use CO2 emission efficiency and emission reduction potential are indicators to analyze the carbon dioxide emission efficiency and emission reduction potential of China’s industry and its sub-sectors. Jiang-Xue Z [38] used the SBM function to study the relationship between environmental regulation and industrial greening. Liang-Wen L [39] uses the DEA method to measure the overall green technology innovation efficiency of industrial enterprises in different regions to analyze China’s industrial green innovation efficiency. From this, it can be seen that in the existing measurement methods of industrial green level, most of them tend to assume that the technical level in the region is the same, and the decision-making units of different technical frontiers are compared under the same conditions. The scientificity and rationality of this method need to be considered without taking into account the regional heterogeneity. Battese, O’Donnell, Chiu et al., Zhang et al [40–43] have confirmed that it is possible to use the common pre-provincial law to determine the production efficiency measurement problem under the heterogeneity of technology.

The second is the sample selection of industrial green production efficiency. With the deepening of the concept of green industrial production, more and more scholars have studied industrial green development in various regions of the world, and Walz R [44] has analyzed industrial green production in newly industrialized countries. Eiadat, Carrión-Flores, Chiou [45–47] assessed the level of industrial green production in Jordan, the United States and Taiwan. Guo Y [48] based SBM-DEA model calculates the green development efficiency of 34 cities in Northeast China, and determines whether industrial agglomeration promotes or hinders the green development efficiency. In the past, there were few studies on the key strategic area of the Yangtze River Economic Zone in the literature, and a few studies on the industrial green production efficiency of the Yangtze River Economic Zone mainly focused on the inter-provincial and industrial levels in China. Regional comparative analysis has just begun. The research at the urban level is still blank, and the lack of accurate identification of the level and source of industrial green development in individual cities cannot reasonably reflect the negative problems caused by industrial development. For example, Da-Ming Y et al concluded that industrial-technological innovation in 11 provinces and cities along the Yangtze River Economic Zone showed obvious regional differences. Xing [49] evaluates the industrial ecological efficiency of the areas along the Yangtze River Economic Zone through inter-provincial total factor productivity. Chuan-Qing WU et al [50] uses the panel data of one province and city along the Yangtze River Economic Zone and uses the SBM model to find that the efficiency of industrial green development in the Yangtze River Economic Zone is in the lower reaches of China’s average level. From the current research results, the literature mostly focuses on the growth of urban total factor productivity, while the research literature on the growth of urban green total factor productivity is still very few. The research on the green total factor of urban industry in the Yangtze River Economic Zone, a national key strategic region, is still blank.

Because this, the contribution of this paper mainly has the following three points: Firstly, from the perspective of research, the environmental factors are introduced into the evaluation system of industrial GTFP of the Yangtze River Economic Zone, and the unexpected output is included to highlight the importance of environmental problems for the industry of the Yangtze River Economic Zone; Secondly, in the research method, considering the regional heterogeneity, the directional distance function model and MML exponent model are constructed to evaluate the industrial GTFP in the upper, middle and lower reaches of the Yangtze River. It is decomposed into technical efficiency EC and technological progress TEC, which provides a scientific reference for realizing the differential improvement of urban ecological efficiency in the Yangtze River Economic Zone. Thirdly, in terms of research data, the city-level data of the Yangtze River Economic Zone from 2006 to 2018 are selected and updated to better fit the development and changes of the Yangtze River Economic Zone. It can more accurately evaluate the changes of green total factor productivity in the Yangtze River Economic Zone in China’s current era. To put forward policy recommendations to improve the green total factor productivity of industry in the Yangtze River Economic Zone of China and the policy recommendations for green development. This paper mainly selects the panel data of 108 cities in the Yangtze River Economic Zone from 2006 to 2018, constructs the direction distance total factor productivity model and MML index to calculate and decompose the industrial GTFP of different cities.

## 3. Method

This paper mainly selects the panel data of 108 cities in the Yangtze River Economic Zone from 2006 to 2018 and constructs the direction distance total factor productivity model and MM index to calculate and decompose the industrial GTFP of different cities.

### 3.1. Directional distance function

Set decision units according to provinces, and each decision unit carries out Z inputs: $x=(x_{1},x_{2},\ldots ,x_{Z})\in R_{+}^{Z}$,Obtain M kinds of expected output: $y=(y_{1},y_{2},\ldots ,y_{M})\in R_{+}^{M}$ and N kinds of unexpected output: $\text{f}=\left({\text{f}}_{1},{\text{f}}_{2},\ldots ,{\text{f}}_{\text{N}}\right)\in {\text{R}}_{+}^{\text{N}}$. Then the input-output combination form of decision making unit is current production possibility set:

$${\text{P}}^{s}(x^{s})=\{(y^{s},f^{s})|x^{s}can\;produce(y^{s},f^{s})\}$$ (1)

Under the condition that the production possibility set Ps of the decision unit meets the condition that the expected output is also zero and the unexpected output can be disposed of, the directional distance function is specifically defined as:

$$\vec{{\text{D}}_{0}}(x,y,f;g_{y,}g_{\partial })=\max\{\beta |(y+\beta_{g_{y}},f-\beta_{g_{f}})\in P(x)\}$$ (2)

Where, β is the directivity distance function value, g = (*g*_*y*_, *g*_*f*_) is the direction vector, generally taken as *g* = (*y*,—*f*) The purpose of using the directional distance function is to obtain the β value when the expected output (y) is maximized and the unexpected output (f) is minimized.

### 3.2. Directional distance DEA model based on meta-frontier

Hayami and Ruttan [51] put forward the concept of "Meta-frontier", which was later widely used by many scholars in the measurement of production efficiency [41]. The DEA-Malmquist exponential model is a common non-parametric model to measure total factor productivity, which can use linear programming method to measure the rate of change of total factor productivity with time. At the same time, the DEA method does not limit the number of output variables and can input multiple input-output variables, which meets the requirements of energy consumption as a production input and environmental pollution as unexpected output when calculating the industrial green total factor productivity. The green total factor productivity index of the Yangtze River Economic Zone is GTFP = 1-β.

From Chiu [43], it can be calculated by the following DEA model:

$$\begin{array}{l} \vec{D_{0}^{g}}(x^{s},y^{s},f^{s};y^{s},-f^{s})=\max\beta^{G}\\ s.t.\left\{\begin{array}{cccc} \sum_{s=1}^{T}\sum_{k=1}^{K_{G}}\mu_{k}^{s}x_{kz}^{s}\geq x_{z}^{s}z=1,\ldots ,\text{Z} & & & \\ \sum_{s=1}^{S}\sum_{k=1}^{K_{G}}\mu_{k}^{s}y_{km}^{s}\geq (1+\beta^{G})y_{m}^{s},m=1,\ldots ,M & & & \\ \sum_{s=1}^{S}\sum_{k=1}^{K_{G}}\mu_{k}^{s}y_{kf}^{s}\geq (1-\beta^{G})f_{n}^{s},n=1,\ldots ,N & & & \\ \mu_{k}^{s}\geq 0;k=1,\ldots ,K_{G};s=1,\ldots ,T & & & \end{array}\right. \end{array}$$ (3)

$$\begin{array}{l} \vec{D_{0}^{g}}(x^{s},y^{s},f^{s};y^{s},-f^{s})=\max\beta^{S}\\ s.t.\left\{\begin{array}{cccc} \sum_{s=1}^{S}\sum_{k=1}^{K_{S}}\theta_{k}^{s}x_{kz}^{s}\geq x_{z}^{s}z=1,\ldots ,\text{Z} & & & \\ \sum_{s=1}^{S}\sum_{k=1}^{K_{S}}\theta_{k}^{s}y_{km}^{s}\geq (1+\beta^{S})y_{m}^{s},m=1,\ldots ,M & & & \\ \sum_{t=1}^{S}\sum_{k=1}^{K_{S}}\mu_{k}^{s}y_{kn}^{s}\geq (1-\beta^{S})f_{n}^{s},n=1,\ldots ,N & & & \\ \mu_{k}^{s}\geq 0;k=1,\ldots ,K_{S};s=1,\ldots ,S & & & \end{array}\right. \end{array}$$ (4)

Where Z, M and N represent the number of input variables, positive outputs (expected outputs) and negative outputs (unexpected outputs), KG and KR represent the number of DMU at the Meta-frontier and regional frontier, respectively. μ and λ are strength variables at both levels. The technology gap rate (TGR) is numerically equal to the ratio of the efficiency of Meta-frontier technology to the efficiency of group frontier technology, reflecting the gap between the actual technical level and the potential optimal technical level. It reflects the deviation degree of the evaluated DMU actual production technology level from the Meta-frontier, this index is a reverse index, the smaller the index value, the greater the gap between the actual production technology and the Meta-frontier technology of DMU.


$$TGR^{\text{s}}\left(x_{s},y_{s}\right)=\frac{MTE^{\text{s}}\left(x_{s},y_{s}\right)}{GTE^{\text{s}}\left(x_{s},y_{s}\right)}=\frac{D_{m}^{s}\left(x_{s},y_{s}\right)}{D_{k}^{s}\left(x_{s},y_{s}\right)}$$ (5)

Further derivation of the TGR considering the unexpected output:

$$0\leq TGR^{\text{s}}\left(x_{s},y_{s},b_{s}\right)=\frac{MTE^{\text{s}}\left(x_{s},y_{s},b_{s}\right)}{GTE^{\text{s}}\left(x_{s},y_{s},b_{s}\right)}=\frac{1/1+D_{m}^{\text{s}}\left(x_{s},y_{s},b_{s};g_{s}\right)}{1/1+D_{K}^{\text{s}}\left(x_{s},y_{s},b_{s};g_{s}\right)}\leq 1$$ (6)

The specific meaning of each indicator is shown in Table 1.

**Table 1:** Meanings of indices.

Index	Variation Tendency	Meanings
GTFP	GTFP = 1	The development of GTFP is getting better and better under the Meta-frontier.
EC	EC→1	Industrial green total factor productivity has the advantage of scale and structure development.
	EC→0	The scale of industrial green total factor productivity needs to be transformed and upgraded.
TC	TC→1	Industrial green total factor productivity has the advantage of technological development.
	TC→0	The technical level of industrial green total factor productivity is relatively low.
TGR	TGR→1	The technical level of the area has reached the potential best.
	TGR→0	There is a large space for the development of technology level in this area.

### 3.3. Meta-frontier Malmquist Luenberger index

In This paper, the industrial green total factor productivity of the Yangtze River Economic Zone is calculated in detail. In order to emphasize the added environmental variables, the Metafrontier-Malmquist-Luenberger index is recorded as the industrial green total factor productivity of the Yangtze River Economic Zone., GTFP.

Pastor and Lovell [52] pointed out that MML can effectively cope with the infeasibility of M exponential linear programming and the inefficiency of traditional Mml Meet the conditions of transfers, additivity, etc. Oh [53] applies the metafrontier method to the global reference Malmquist model to construct the Metafrontier-Malmquist-Luenberger exponential method to overcome the above defects. Therefore, it is possible to analyze the production potential of the period by encircling together to construct the global frontier, which is expressed as: $Y^{G}(x)=Y^{1}(x^{1})\cup Y^{2}(x^{2})\ldots Y^{S}(x^{S})$, and analyze the change of GTFP from the global point of view.

The Metafrontier-Mesquist-Luenberger index consists of the distance between the two adjacent production points and the Meta-frontier. The problem of arbitrary selection is avoided and the model is transitive. GTFP represents the industrial green total factor productivity index of the Yangtze River Economic Zone under the Meta-frontier. The index is greater than 1, indicating an increase in industrial green total factor productivity in the Yangtze River Economic Zone. According to Wang et al [54], we can decompose the Metafrontier-Malmquist-Luenberger (MML) index with negative output into the following formula:

$$MML_{\text{s}}^{s+1}=MEC_{\text{s}}^{s+1}\times MTC_{s}^{s+1}$$ (7)

$$=\left\{\left[\frac{1+{\vec{D}}_{m}^{\text{s}}\left(x_{s},y_{s},b_{s};g_{s}\right)}{1+{\vec{D}}_{m}^{s+1}\left(x_{s+1},y_{s+1},b_{s+1};g_{s+1}\right)}\right]\times \sqrt{\left[\frac{1+{\vec{D}}_{m}^{s+1}\left(x_{s},y_{s},b_{s};g_{s}\right)}{1+{\vec{D}}_{m}^{\text{s}}\left(x_{s},y_{s},b_{s};g_{s}\right)}\times \frac{1+{\vec{D}}_{m}^{s+1}\left(x_{s+1},y_{s+1},b_{s+1};g_{s+1}\right)}{1+{\vec{D}}_{m}^{\text{s}}\left(x_{s+1},y_{s+1},b_{s+1};g_{s+1}\right)}\right]}\right\}$$

## 4. Index setting and sample selection

### 4.1. Selection of indicators

Green total factor productivity reflects the coupling and coordinated development level of resource factor input, economic development level and ecological pollution load. According to the connotation of GTFP, 108 cities are selected as decision-making units, combined with the characteristics of industry in the Yangtze River Economic Zone, and related research results are used for reference and improvement. Select four input indicators and two output indicators to build the green total factor productivity evaluation index system as follows.

1. Capital investment. The total amount of current assets investment in industries above the scale of the Yangtze River Economic Zone represents the capital stock. The Capital investment reflects the direct or indirect total capital investment in order to serve industrial production, but because there is a higher correlation between the current assets of manufacturing companies and corporate performance, Qiu Y. Qiao D [54], therefore, current assets are selected to estimate the capital investment of each province and city. Through the analysis of the indexes, it is found that there is no need to exclude the price factors when evaluating the actual level and development of economic benefits.
2. A number of employees. The number of industrial employees in 108 prefecture-level cities in the Yangtze River Economic Zone represents labor input. In This paper, the data of industries above the scale are mostly used in the selection of industrial indicators, so we use the practice of Yue Hongfei et al. [55] for reference. The annual average number of workers employed in industries above the scale is used as the index of labor input.
3. Power consumption. It reflects the resource input together with the industrial water consumption above the scale.
4. Water consumption.
5. Total profit. This is the expected output, and according to Nasution Z [56], we directly use the total profit as the positive output of the gross industrial output of each city.
6. Discharge of industrial wastewater.
7. Emission of industrial solid smoke and dust.
8. Industrial-sulfur dioxide emissions. These three parts are regarded as unexpected outputs. Based on the treatment method of Liu K, Lin B [57], the direct right method is used to objectively allocate the emission of industrial wastewater, industrial solid smoke, and industrial sulfur dioxide in Mingcheng City. And then build a pollution index as an unexpected output.

Descriptive statistical analysis of inputs and outputs is shown in Table 2:

**Table 2:** Descriptive statistics of input and output indicators.

Criterion Layer	Index	Unit	Group	Max	Min	Mean	Std.Dev
Input	Current Assets	Billion	Upper Reach	94890957	83260	7141515.93	12492415.07
			Middle Reach	71146801	90288	6195398.85	9475612.97
			Lower Reach	255868131	283571	23288975.22	36572951.69
			Total	255868131	12956197.56	12956197.56	25443330.56
	Employees	10 Thousand	Upper Reach	429.13	0.66	22.76	48.49
			Middle Reach	105.3	1.51	19.94	18.03
			Lower Reach	273.47	0	39.39	47.34
			Total	429.13	0	28.13	41.36
	Water Consumption	Billion	Upper Reach	71068	101	5512.45	10659.69
			Middle Reach	80662	222	7438.31	11687.89
			Lower Reach	256236	385	16647.16	34281.04
			Total	256236	101	10381.47	23415.89
	Power Consumption	Billion	Upper Reach	6536703	1016	422149.78	773270.71
			Middle Reach	2796653	4510	340442.69	394265.29
			Lower Reach	12277696	17136	1015501.08	1561286.48
			Total	12277696	1016	620167.81	1115446.3
Positive Output	Profit	Billion	Upper Reach	6536703	1016	422149.78	73270.71
			Middle Reach	2796653	4510	340442.69	394265.29
			Lower Reach	12277696	17136	1015501.08	1561286.48
			Total	12277696	1016	620167.81	1115446.3
Negative Output	Tri-wastes	Billion	Upper Reach	0.016	0.008	0.009	0.001
			Middle Reach	0.01	0.008	0.009	0.0004
			Lower Reach	0.014	0.008	0.009	0.0011
			Total	0.016	0.008	0.0092	0.00099

### 4.2. Data sources

This paper mainly selects the industrial production panel data of 11 industrial production panels in the Yangtze River Economic Zone from 2006 to 2018, constructs the direction distance total factor productivity model and MML index to calculate and decompose the industrial green total factor productivity in different regions. The original data used in this paper are all from China Urban Statistical Yearbook, all economic indicators are adjusted based on 2006, and the data missing of individual years are replaced by weighted average calculation results. "Three wastes" emission mainly includes industrial emissions, considering the integrity and availability of data, so the industrial "three wastes" emission data is selected to approximate the industrial unexpected output.

### 4.3. Sample selection

**Fig 1:**
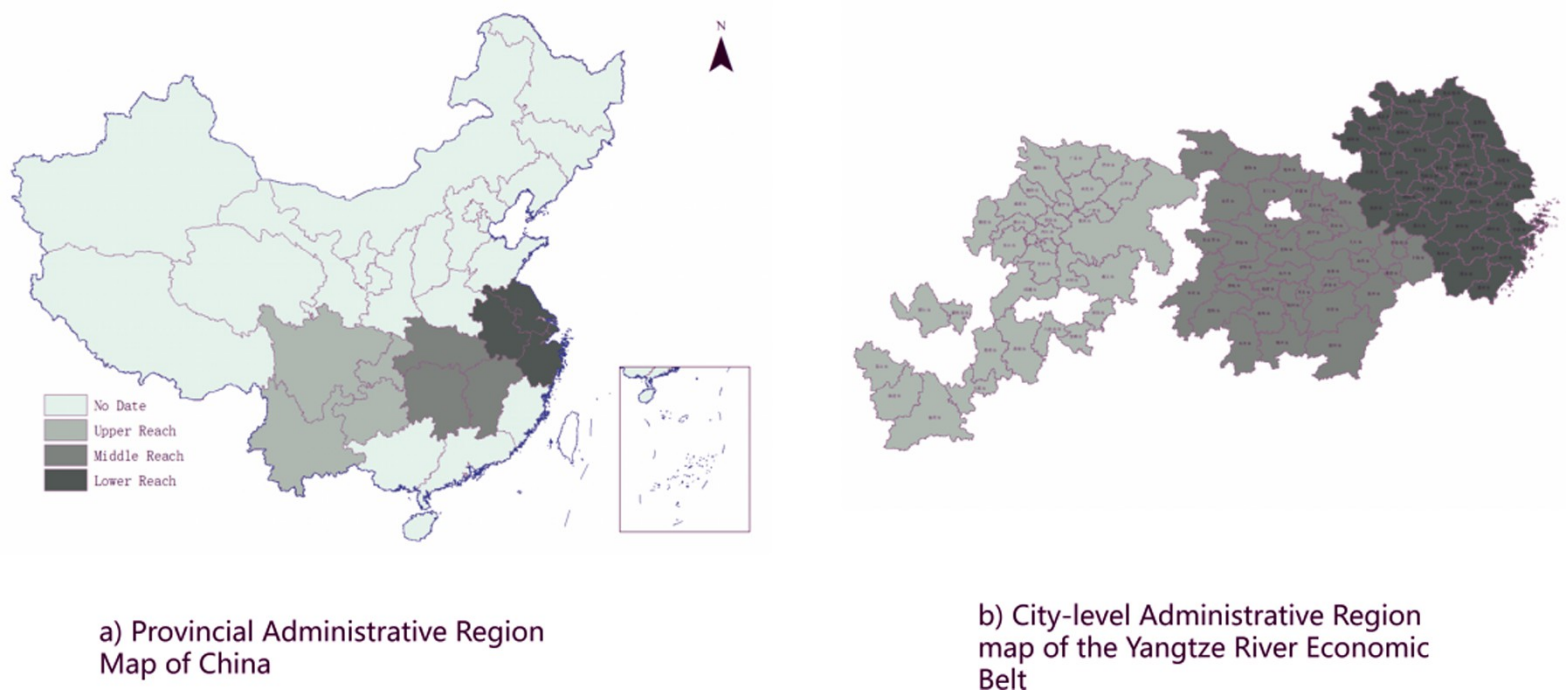
Classification of the prefecture-level cities in the Yangtze Econogic Belt.

Given the problems of sustainable industrial development in the Yangtze River Economic Zone, the factors such as resource carrying capacity, environmental capacity, ecological type, and development basis of the Yangtze River Economic Zone are considered comprehensively. As shown in Fig 1, in the main stream of the Yangtze River, the upper reaches of Yichang City in Hubei Province are above, the middle reaches are from Yichang to Hukou County in Jiujiang, Jiangxi Province, and the lower reaches are below Hukou County.

The specific grouping is shown in Table 3. This paper takes the prefecture-level and above cities in the Yangtze River Economic Zone as the research object, because Bijie City and Tongren City are newly established prefecture-level cities at the end of 2011, resulting in data incoherence, so they are eliminated. In addition, since the data of urban environmental pollution emissions began to be counted in 2006, the research time dimension of this paper is determined to be from 2006 to 2018. Moreover, this period is also a period of rapid expansion of urban scale in the Yangtze River Economic Zone. Choosing this time period will help to further understand the quality of urban development in the Yangtze River Economic Zone during the period of rapid urban development.

**Table 3:** City grouping of Yangtze River Economic Zone.

Regional groupings	Province	City
**Upper Reaches**	Sichuan Province	Chengdu, Mianyang, Zigong, Panzhihua, Luzhou, Deyang, Guangyuan, Suining, Neijiang, Leshan, Ziyang, Yibin, Nanchong, Dazhou, Ya’an, Guang’an, Bazhong, Meishan
	Yunnan Province	Kunming, Qujing, Yuxi, Zhaotong, Baoshan, Lijiang, Pu’er, Lincang
	Chongqing Municipality	Chongqing
	Guizhou Province	Guiyang, Zunyi, Anshun, Liupanshui
**Middle Reaches**	Hubei province	Wuhan, Huangshi, Xiangyang, Jingzhou, Yichang, Shiyan, Xiaogan, Jingmen, Ezhou, Huanggang, Xianning, Suizhou
	Jiangxi Province	Nanchang, Jingdezhen, Pingxiang, Jiujiang, Xinyu, Yingtan, Ganzhou, Ji’an, Yichun, Fuzhou, Shangrao
	Hunan Province	Changsha, Zhuzhou, Xiangtan, Hengyang, Shaoyang,Y ueyang, Changde, Zhangjiajie, Yiyang, Chenzhou, Yongzhou, Huaihua, Loudi
**Lower Reaches**	Shanghai Municipality	Shanghai
	Zhejiang Province	Hangzhou, Ningbo, Wenzhou, Jiaxing, Huzhou, Shaoxing, Jinhua, Quzhou, Zhoushan, Taizhou, Lishui
	Jiangsu Province	Nanjing, Wuxi, Xuzhou, Changzhou, Suzhou, Nantong, Lianyungang, Huaian, Yancheng, Yangzhou, Zhenjiang, Taizhou, Suqian
	Anhui Province	Hefei, Wuhu, Bengbu, Huainan, Ma’anshan, Huaibei, Tongling, Anqing, Huangshan, Chuzhou, Fuyang, Suzhou, Lu’an, Bozhou, Chizhou, Xuancheng

## 5. Empirical research and analysis

The Yangtze River Economic Zone is long and narrow, and there are great differences in resource endowment and industrial structure among the upper, middle, and lower reaches, so the problem of temporal and spatial heterogeneity should be fully considered when analyzing the green total factor production efficiency of the Yangtze River Economic Zone. In This paper, the Mml index is used to measure the overall temporal and spatial differences of green total factor production efficiency in the Yangtze River Economic Zone from 2006 to 2018, and the three groups are exponentially decomposed. And then reveal the size and source of the difference.

### 5.1. Dynamic analysis of green total factor productivity in Yangtze River Economy and Industry

**Fig 2:**
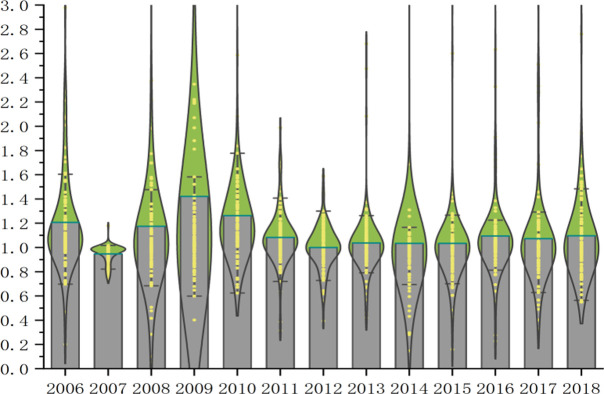
Green total factor productivity of industry in the Yangtze River Economic Zone, 2006–2018.

**Fig 3:**
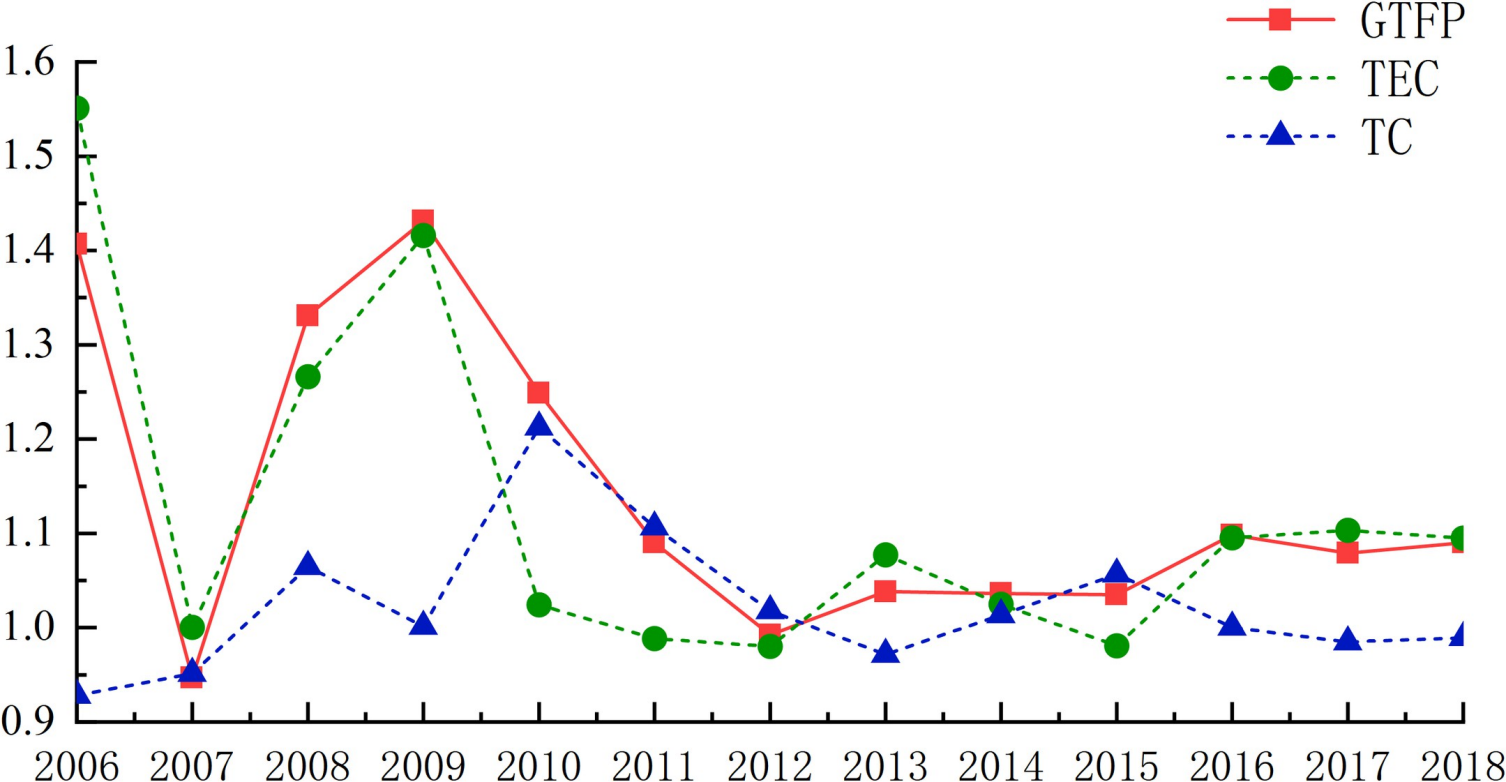
2006–2018 industrial green total factor productivity and its index decomposition.

Figs 2 and 3 describe the changes of green total factor MI and technical efficiency EC and technological progress in 108 cities at prefecture level and above in the Yangtze River Economic Zone from 2006 to 2018. Since 2006, the cities in the Yangtze River Economic Zone have achieved rapid productivity growth and efforts to reduce the cost of economic growth, and the relationship between urban economic growth and resources and the environment has become more coordinated. From 2006 to 2018, the green total factor productivity of the Yangtze River Economic Zone increased by 4.58% annually, indicating that the urban green total factor productivity of the Yangtze River Economic Zone in this period generally showed the characteristics of growth.

From 2006 to 2018, the green total factor productivity of the Yangtze River Economic Zone fluctuated greatly. From 2006 to 2016, green total factor productivity was affected by the growth of technological progress, and there was positive growth. The "Yangtze River Economic Belt Strategy" was upgraded to China’s national strategy in 2015(General Office of the State Council). Starting from 2015, with the continuous expansion of the scale of foreign direct investment, foreign enterprises have brought relatively advanced technology and management experience into the Chinese market, thus promoting the overall technological progress of the industry. The growth rate of green total factor productivity reached a peak of 20.46% in 2015 due to the influence of innovation investment and the active introduction of foreign advanced technology and production equipment in 2005. According to the research of the frontier research group of China’s economic growth, the effect of learning by doing is inverted U-shaped, and China was in the stage of a gradual decline of the effect of learning by doing, while the mechanism of independent innovation has not yet formed. From the decomposition of green total factor productivity, the annual green technology progress index and technology efficiency index during the study period is greater than 1. From the overall trend, green total factor productivity is basically consistent with the trend of green technological progress and scale efficiency, which further verifies that the improvement of green total factor productivity mainly comes from technological progress and the improvement of technological efficiency. At that time, it was in the initial stage of scale effect of urban industrial agglomeration, the speed of resource consumption had not exceeded the speed of resource regeneration and environmental carrying capacity, and environmental pollution and excessive consumption of resources had not restricted the development of green economy. However, in 2017, due to the negative effects of industrial agglomeration, the negative externalities such as excessive rise in land costs and environmental pollution may become increasingly serious. Then it produces a "congestion effect" on technological innovation, which limits the promotion of urban GTFP, so the MI index of this year is less than 1. In April 2018, General Secretary Xi Jinping gave important instructions on the protection of the ecological environment in the Yangtze River Economic Zone, and the state attached great importance to the protection of the ecological environment in the Yangtze River Economic Zone. The Outline of the Development Plan for the Yangtze River Economic Zone was promulgated and implemented, which clearly defined the overall strategy of ecological priority and green development of the Yangtze River Economic Zone, and promoted the development of green factor production in the Yangtze River Economic Zone. In 2018, the growth rate of industrial green total factor productivity in the Yangtze River Economic Zone reached 10.22%.

Based on Malmquist Luenberge index, urban green total factor productivity is generally fluctuating and rising, with an average annual growth rate of 4.58%. Technological efficiency and technological progress are on the rise as a whole, with the average annual growth rate of technological efficiency EC being 7.38% and that of technological progress TEC being 4.06%. The growth of technological efficiency is greater than that of technological progress. It can be concluded that the overall change of green total factor productivity is mainly due to the change of technological progress, which confirms that technological progress is the source of economic efficiency improvement.

From 2006 to 2012, the improvement of green total factor productivity mainly came from the role of technological progress, and after 2012, the role of technological progress began to be further highlighted. The role of technological progress in regional green total factor productivity is becoming more and more obvious. This is in line with the time for China to implement the innovation-driven development strategy, which shows that the role of innovation-driven development strategy can be shown in the Yangtze River Economic Zone. Driven by the national policy of energy saving and emission reduction, the performance of industrial green development in the Yangtze River Economic Zone has been improved to a certain extent, that is, in 13 years, the industry in the Yangtze River Economic Zone has passed low energy consumption and carbon emissions. This is in line with the Yangtze River Economic Zone’s high-quality development environment, promoting the research and development of green industrial products and promoting the application of new technologies in industry. Promoting the transformation of industrial color is closely related. However, it is worth noting that although the overall industrial green total factor productivity in the Yangtze River Economic Zone has increased, the increase is not large, which to some extent shows that the extensive development model leads to the slow industrial green development in the Yangtze River Economic Zone.

From 2006 to 2018, the technological efficiency EC index and the technological progress TEC index of the Yangtze River Economic Zone fluctuated and changed, and there was a significant trend of differentiation. The fluctuation trend of EC index is almost synchronized with MI index, while the fluctuation trend of TEC index is slightly worse than MI index, which shows that the industrial technology efficiency of the Yangtze River Economic Zone is constantly improving. With a good growth effect, technological efficiency has become the main driving force for the growth of industrial green gold factor productivity in the Yangtze River Economic Zone. The decline of technological progress and the weakening of catch-up effect may lead to the widening gap of industrial green total factor productivity among provinces in the Yangtze River Economic Zone, which also indicates that there is still much room for improvement of technological progress. He also said that the industry in the Yangtze River Economic Zone has the problems of low technological progress and poor innovation, which may lead to poor growth of technological progress. It can be inferred that technological efficiency has begun to release the growth effect in the process of continuous accumulation, digestion and absorption, and industrial green total factor productivity has been moving to the forefront of production. However, under the pressure of technological efficiency, the catch-up effect has not been fully released, and the catch-up effect has weakened. Therefore, the industry in the Yangtze River Economic Zone must adhere to innovation and development, pay attention to industrial technology investment and R & D, and promote technological progress and technological efficiency.

### 5.2. Spatial dynamic analysis of urban total factor productivity in the Yangtze Economic Zone under Different Groups

**Fig 4:**
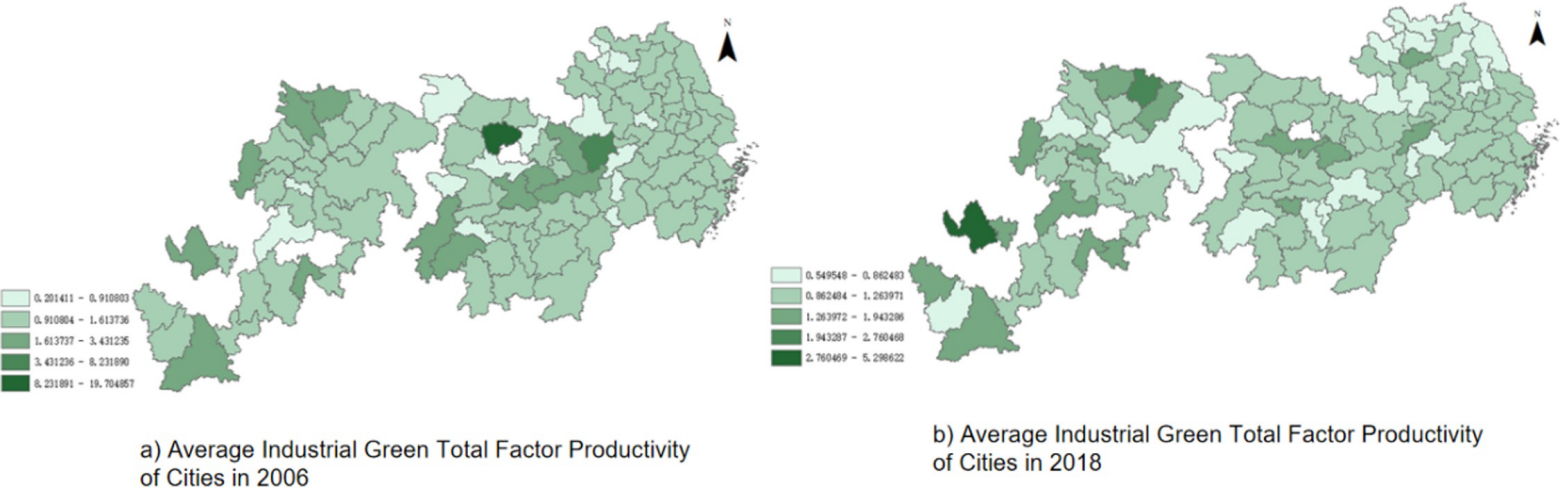
Average green total factor productivity of cities in 2006 and 2018.

Grouping according to different regional development models, and the descriptive statistical results of input and output variables in each region are obtained as shown in Fig 4 below. From Fig 4, we can see that in the input variables (labor, capital, energy, water resources) and output variables (expected output, unexpected output), the green total factor productivity is obtained. In 2006, the average value of the middle reaches was significantly higher than that of the upper and lower reaches, and the average value of the input-output variables of the upper reaches in 2018 was also higher than that of the lower and middle reaches. It shows that there are obvious differences among the three regional development model groups, which shows that it is reasonable to take the regional development model as the basis of group division.

**Fig 5:**
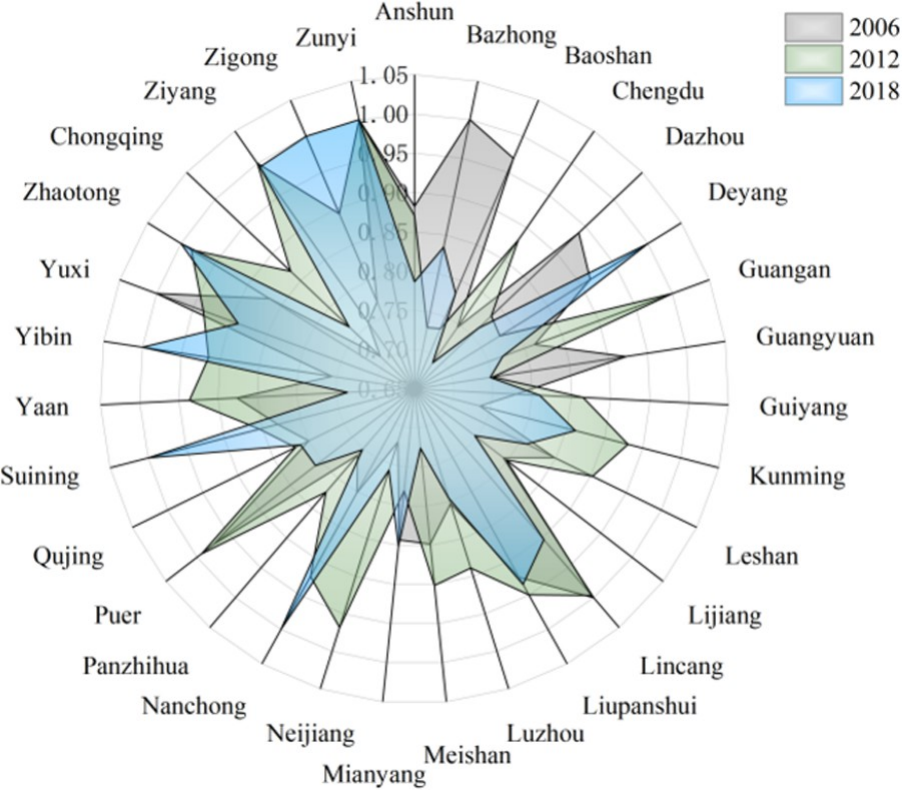
Technology gap rate of cities in the upper reaches.

**Fig 6:**
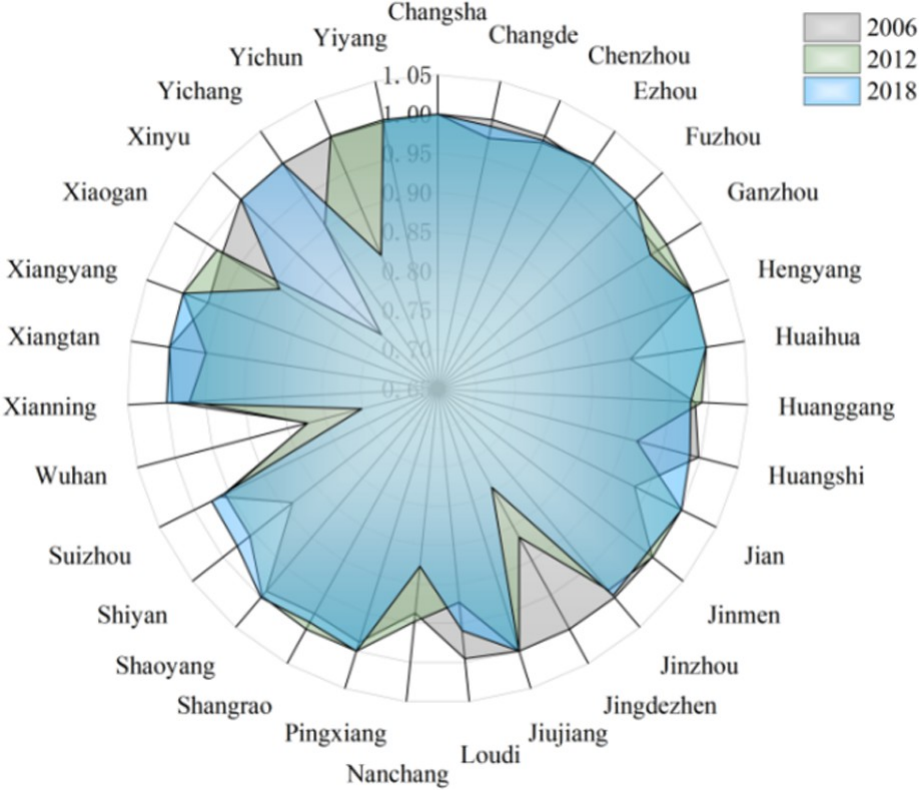
Technology gap rate of cities in the middle reaches.

**Fig 7:**
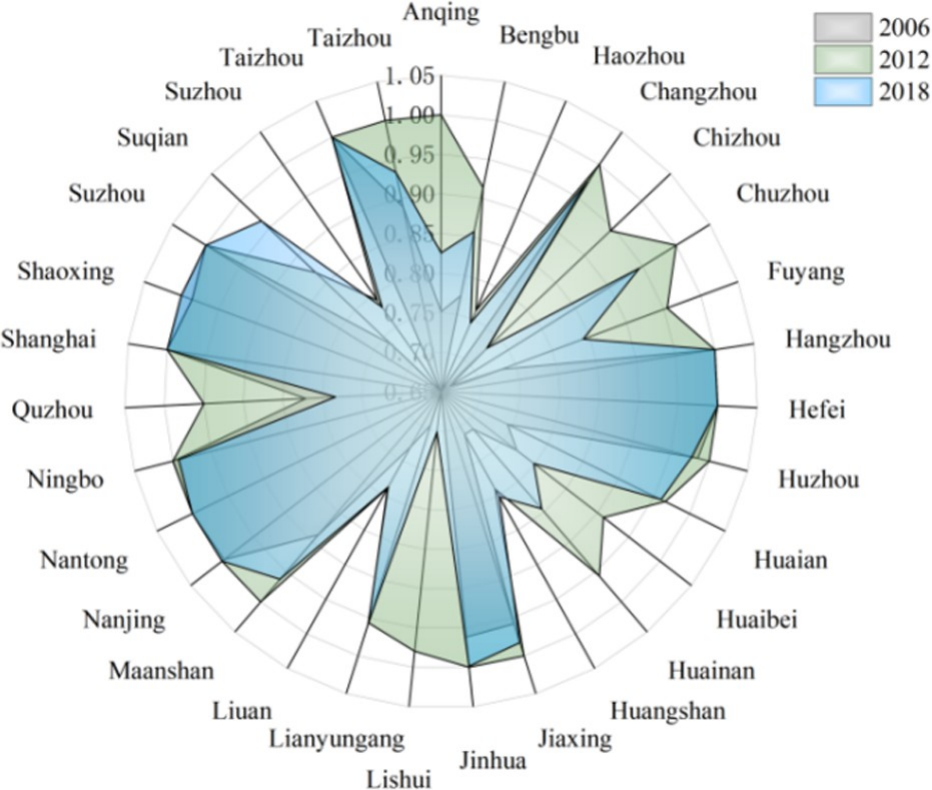
Technology gap rate of cities in the lower reaches.

From 2006 to 2018, the TGR of 108 cities in the upper, middle and lower reaches of the Yangtze River Economic Zone under the Meta-frontier is shown in Figs 5–7.

Specifically, from the perspective of cities in the group:

1. In the upstream urban agglomeration, the technology gap rate of Zunyi City and Yuxi City is higher, while the technology gap rate of Qujing City is lower. The average annual technology gap rate of Zunyi City is 0.959, which is higher than the upstream average (0.828) by 15.8%. Zunyi City makes full use of the advantages of traditional industries, convenient and fast logistics, and transportation, and focuses on the support of coal, wine, electricity, building materials, chemical industry, metallurgy, non-ferrous industries. With the goal of stabilizing production and expanding production capacity, we have cultivated and approved traditional industrial enterprise groups promoted the transformation and upgrading of traditional industries, and achieved remarkable results in accelerating the growth of traditional industries. The annual average technology gap rate of Yuxi City is 0.893, which is 7.8% higher than the upstream average (0.828). Relying on Yukun, Xianfu, and Xinxing Iron and Steel, Yuxi City has built an important iron and steel production base in Yunnan Province, a famous national cigarette industry base relying on Hongta Group, and a world-class led substrate production base relying on Blue Crystal Science and Technology. Wanlu has become the leader of the national aloe industry, Linyuan has become the largest producer of borneol in the country, and Watson is the first phalanx in the national seedling industry. A number of excellent enterprises have continuously injected momentum into the development of Yuxi industry. In the process of industrialization, Qujing City has shown a good trend in general, but there are also a backward industrial base and a low proportion of high-tech industries. The growth mode is extensive and the environmental protection problem is serious. Low risk resistance, unreasonable structure, and other issues.
2. In the middle reaches of the urban agglomeration, Pingxiang and Shaoyang have the highest technology gap rate, and Pingxiang’s TGR is also the highest in the whole Yangtze River Economic Zone, reaching 0.999. Shaoyang reached 0.996. The technical gap rate in Wuhan is the lowest, which is 0.774. The proportion of land and resources in the middle reaches of the region is its unique advantage, accounting for 10.7% of the land area, but it has 30% of the mineral resources, which also creates the conditions for some provinces in the central region to become heavy industry bases. Among them, Pingxiang City is a prefecture-level city in the western region of Jiangxi Province, located at the junction of Hunan and Jiangxi, along the main traffic artery of Shanghai-Kunming Railway, and in the middle of Zhuzhou City and Xuanchun City. Therefore, in the radiation area of Changsha-Zhuzhou-Xiangtan urban agglomeration, it is an important member of the urban agglomeration in the middle reaches of the Yangtze River, and the high-tech gap rate of Pingxiang City benefits from its unique location advantages and extensive transportation network. Insufficient openness is the main restrictive factor for Wuhan’s development. The lack of opening process and low level of opening up lead to the relatively slow development of Wuhan, the long-term failure of institutional mechanisms to lift the shackles, the low-level operation of industrial development, and the relative lag of environmental improvement. Wuhan is an old industrial base of the country, with a heavy industrial structure, a relative shortage of energy resources, high energy consumption, low energy utilization and heavy pollution. In order to realize the leap-forward development of Wuhan and basically realize modernization, we must break the bottleneck constraints of resources and environmental problems on the overall development of Wuhan. Over the years, while promoting rapid economic growth in Wuhan, the work of resources and environment has also achieved certain results. In 2006, the reuse rate of industrial water reached 77%. Because the space for energy saving and consumption reduction of high energy-consuming enterprises is relatively limited, the pressure of energy saving and emission reduction in Wuhan is very great. Wuhan is facing the urgent task of vigorously developing low energy consumption industries and speeding up the adjustment of industrial structure. As an important force to promote China’s economic growth, the sound development of Hubei Province is particularly important. In 2004, the central government put forward the strategy of the rise of central China. The strategy points out that we should strive to achieve a significant economic improvement in the central region by 2015. Therefore, the central region seizes the opportunity advantage of national policy orientation and takes advantage of the opportunity of undertaking industrial transfer in the eastern region to contribute more and more to the total national economy.
3. In the downstream urban agglomeration, Ningbo has the highest technology gap rate, and also the highest in the Yangtze River Economic Zone, reaching 0.999. Haozhou is the lowest, 0.729. By the end of 2016, the proportion of R & D investment of industrial enterprises above designated size in the main business income of Ningbo will reach 1.5%, and the number of invention patents authorized by 10000 people will exceed 4. Average annual growth of 10%; The total industrial output was 1050 billion yuan, with an average annual growth of 11%. It has gathered about 1000 R & D institutions, 100 innovation platforms and exceeded trillion yuan in total industrial output value. Ningbo has initially built a strong industrial city, becoming a demonstration area for the construction of a strong industrial city in the whole province, a pioneer area for the transformation and upgrading of the national industry, and an important international advanced festival manufacturing base. The structural contradictions of industry in Haozhou City are prominent, and the proportion of traditional industries is too large. The main body of Haozhou’s industrial economy is the middle and low technology industry, the absolute proportion of traditional industries, and the slow development of high-tech industries. Backward technology, aging equipment, insufficient investment in technology and slow pace of transformation are the practical problems facing the industrial transformation and upgrading of Haozhou. Although Haozhou has made great efforts to upgrade and transform traditional industries in recent years, traditional industries still occupy an absolute position in the industrial economy, and high-tech enterprises are almost zero. From the perspective of product composition, the proportion of traditional industrial products is large, while the proportion of high-tech products is small. The proportion of low-grade products is large, the proportion of middle and high-grade products is small, and there are almost no high-tech products in line with the world development trend.

**Fig 8:**
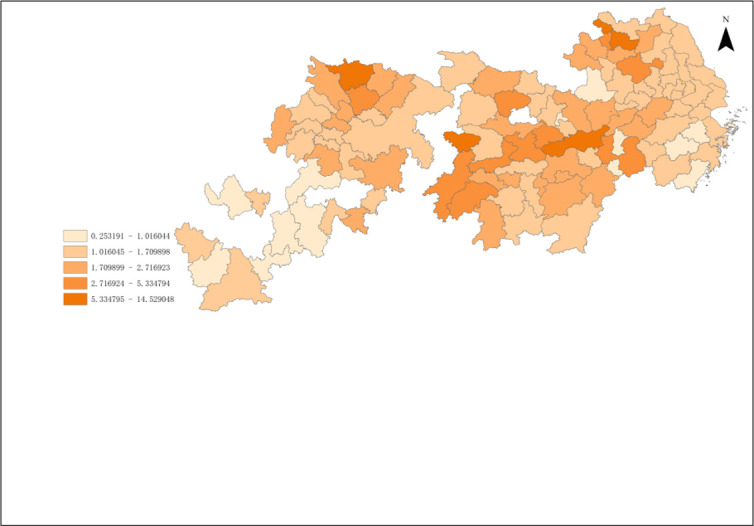
Green total factor productivity of cities in Yangtze River Economic Zone.

**Fig 9:**
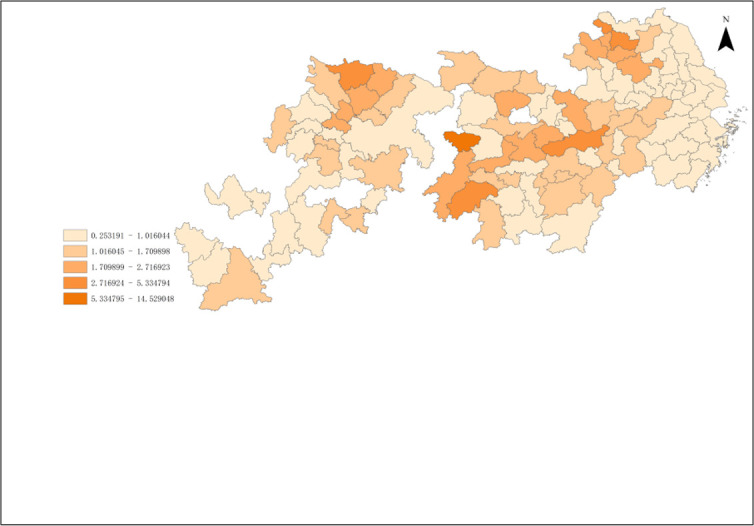
Industrial technical efficiency of cities in Yangtze River Economic Zone.

**Fig 10:**
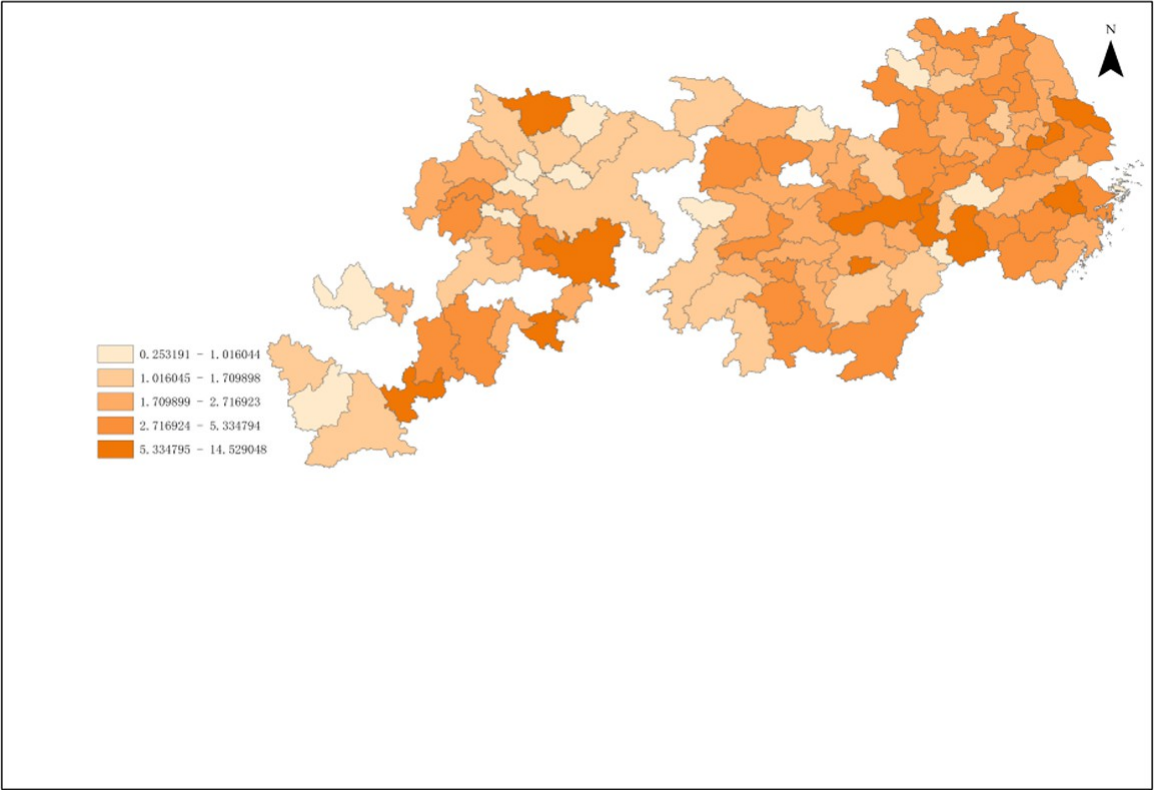
Industrial technology progress of cities in Yangtze River Economic Zone.

According To the results of Malmguist model method, the annual average Malmquist index of industrial green development efficiency of cities in the Yangtze River Economic Zone from 2006 to 2018 and its decomposition items EC and TC can be obtained. As shown in Figs 8–10.

From Fig 8, it can be obtained that the production efficiency value of industrial green total factors in the Yangtze River Economic Zone from high to low in 2006–2018 is upstream, midstream, and downstream in turn. The annual growth rate of green total factor productivity was the highest in the upstream region, which was 5.39%. The average annual growth rate of green total factor productivity in the middle reaches was 5.33%. The annual growth rate of green total factor productivity in the downstream region was the lowest, which was 3.02%. The total factor productivity of industrial green in the upper and middle reaches of China is similar, but both of them are higher than that in the lower reaches.

From the color depth of Fig 8, it can be seen that the green total factor productivity of 11 provinces and cities in the Yangtze River Economic Zone from 2006 to 2018 roughly presents certain spatial agglomeration characteristics. It shows a certain spatial effect or urban agglomeration effect, but there are great differences among the internal regions, and the cities with a higher production efficiency of green total factors are adjacent. The spatial agglomeration effect of 11 provinces and cities in the Yangtze River Economic Zone can be roughly divided into three categories: Shanghai, Jiangsu, The agglomeration effect of the upper reaches of the Yangtze River represented by 41 prefecture-level cities in Anhui Province and Zhejiang Province; The agglomeration effect of the middle reaches of the Yangtze River represented by 36 prefecture-level cities in Hubei, Hunan and Jiangxi provinces; the agglomeration effect of the lower reaches of the Yangtze River represented by 31 prefecture-level cities in Chongqing, Sichuan, Yunnan and Guizhou provinces.

The proportion of industrial green total factor productivity in the upper reaches of the Yangtze River has exceeded 33.3%. The green total factor productivity at the junction of Sichuan, Yunnan and Guizhou in the upper reaches of the Yangtze River is relatively high, which has become a spatial connection channel and core radiation driving area with the upper reaches. The production efficiency of green total factors in Sichuan is gradually improving, which may become the core growth pole in the upper reaches of the Yangtze River. The industrial green productivity in the middle reaches of the Yangtze River is relatively high, accounting for a medium proportion, and has been hovering around 33.5%, which is the stable area of the Yangtze River Economic Zone. On the whole, the efficiency of green total factor Shengjiang River in Hunan and Jiangxi provinces in the middle reaches of the Yangtze River is higher. Specifically, due to geographical proximity and accessibility of transportation, the proportion of green total factor productivity in Hunan and Jiangxi provinces has increased year by year, which has become an important link between the Yangtze River Delta and the middle reaches of the Yangtze River. The proportion of green total factor productivity in Hunan Province has exceeded 10%, which has become an important core growth pole of industrial green development in the middle reaches of the Yangtze River, but the spatial links with the Yangtze River Delta and the upper reaches of the Yangtze River are generally weak. This may be closely related to Hunan Province’s vigorous promotion of the strategy of "building the middle triangle and building the fourth pole". In recent years, Hunan and Hubei, Jiangxi and Anwei have comprehensively promoted the construction of urban agglomeration in the middle reaches of Changgong from the aspects of top-level promotion, institutional mechanism establishment and department cooperation, which has led to the continuous strengthening of the links between Hunan and the three provinces in the middle reaches of the Yangtze River, while the radiation acceptance of the Yangtze River Delta and the Yangtze River The green total factor productivity of the Yangtze River Economic Zone shows a more obvious "ladder" trend from the lower reaches to the upper reaches, which may be related to the fact that most of the lower reaches of the Yangtze River are located in the coastal open areas, which is in the critical period of industrial transformation, and some cities are based on their own technological level. The lower reaches of the Yangtze River is an important base for the green development of industry and an advantageous agglomeration area in the Yangtze River Economic Zone, and is also the core of China’s "T-shaped" development strategy in the Yangtze River Delta region. The industrial development is relatively early, the regional transportation is convenient, the inter-provincial cooperation is in-depth, and the production of people, logistics and information flow is important It should also promote the Yangtze River Delta region to become an active area of industrial economic development in the Yangtze River Economic Zone, and develop the Yangtze River Delta region as the core growth pole of industrial green development in the Yangtze River Economic Zone. Shanghai is an international financial and technological innovation center, which has strong advantages in innovation investment and financial development, providing financial and intellectual support for enterprises to develop clean technology, and promoting green transformation and clean production substitution. It is worth noting that Shanghai, as the green development and growth pole of industry in the Yangtze River Economic Zone, has significant advantages, which may be limited by location distance and traffic obstacles, resulting in Shanghai not showing strong agglomeration effect and diffusion effect in the Yangtze River Economic Zone.

According to the data in Fig 9, we can see that Chongqing and Sichuan, which have higher total factor productivity, have higher technological efficiency. Yunnan, the lowest, has the lowest technical efficiency, so it should further improve the technical efficiency. During the research period, the technical efficiency of all provinces and cities has been improved and increased at an average annual growth rate of 7.38%. Among them, Guizhou and Hunan have the fastest promotion speed. The existing scale system of these provinces and municipalities needs to be further improved. The technical efficiency is "the middle reaches > the upper reaches > the lower reaches > the lower reaches", and the technical efficiency of the lower reaches is close to 1, indicating that the technical efficiency dividend of the lower reaches is disappearing, while the cities of the upper reaches are in the rising period of the technical efficiency dividend.

From the point of view of decomposition indicators, the efficiency of technological progress plays a decisive role in the green development of industry total factor productivity, science and technology is the first productive force, economic growth theory and a large number of empirical studies show that technology is the source of growth of total factor productivity. And from the decomposition effect of time dynamic analysis, we can also know that the technical effect is the main factor affecting the change of green total factor productivity, especially under the condition of Hicks neutral. Green technology progress is green total factor productivity. Modern economic growth theory holds that the level of scientific research technology can improve the utilization rate of factors and the level of management technology, thus promoting the growth of green total factor productivity. According to the data in Fig 10, we can see that the middle and lower reaches of the Yangtze River have high technical efficiency. Shanghai’s industrial technological progress ranks in the middle of the Yangtze River Economic Zone, ranking 16th (0.342), while the technological efficiency index ranks in the lower reaches, indicating that Shanghai’s industrial technological progress promotes industrial green productivity to be approaching the frontier of production. However, with the continuous increase of the scale of traditional resource input factors, the redundancy of resource input factors leads to the decline of marginal productivity of resources, which leads to the decline of technological efficiency. Sichuan’s industrial technological progress index is low, while the technological efficiency index is greater than 1, indicating that its industrial green hair mainly depends on the catch-up effect brought by technological efficiency, while the growth effect brought by technological progress is weak. Anhui, Jiangxi, Hubei, Hunan, Chongqing and other provinces and cities have higher industrial technology progress index (all greater than 1), while the technology efficiency index is low, indicating that the green development of industry in these provinces is realized by the catch-up effect brought about by the growth effect brought about by technological progress. The growth of industrial green productivity in these provinces and cities shows that the technological progress and technological efficiency of industrial development in the middle and upper reaches of the Yangtze River have the advantage of backwardness, especially under the support of the policy system of the rise of the central region and the continuous promotion of the strategy of the western development. The technical and resource elements of industrial development have flowed into the central and western regions, thus improving the performance of industrial green development in the middle and upper reaches of the Yangtze River. The main reason for the green productivity of industry in Jiangsu and Zhejiang provinces is that the growth effect of industry is basically completed, while the catch-up effect is not obvious. Specifically, the continuous increase of the scale of resource input in the two provinces may lead to the change of false technological efficiency driven by excessive dependence on factors, or it may be that the utilization efficiency level of existing resource elements will encounter bottlenecks when it reaches a certain level. This led to the factor curse, resulting in the two provinces to reduce the importance of technical efficiency. It can be inferred that industrial green development is not a simple problem of technological progress, and technological efficiency is also the proper meaning of promoting industrial green productivity growth. The green development of industry in Guizhou and Yunnan provinces is in the backward position of the Yangtze River Economic Zone, which may be due to the rising period of scale dividend in Guizhou and Yunnan provinces, and pay more attention to the technical efficiency of industrial green development. However, due to the development concept, technical support, social economy and other factors, the industrial technological innovation ability is low and the degree of self-credence is not high. In addition, the quality of human resources in the two provinces is not high, resulting in low technological progress index, which negatively offsets the catch-up effect brought about by technological efficiency improvement, and ultimately affects the green development of industry in the two provinces.

## 6. Conclusions and policy recommendations

Based on the DEA model, this paper constructs the Mml index considering the expected output and the unexpected output, and calculates the GTFP of China from 2006 to 2018. The conclusions are as follows:

1. During the sample period, the industrial green total factor productivity in the Yangtze River Economic Zone shows the spatial characteristics of differential growth and the temporal characteristics of volatility growth. It shows the fluctuation characteristics of the “N” shape. The industrial green total factor productivity in the Yangtze River Economic Zone generally fluctuated and increased, with an average annual growth rate of 4.58%/o. Since the sample calculation period, the cities in the Yangtze River Economic Zone have achieved rapid productivity growth and efforts to reduce the cost of economic growth, and the relationship between urban economic growth and resources and environment has become more coordinated. From 2006 to 2016, the green total factor productivity of urban industry in the Yangtze River Economic Zone generally showed the characteristics of growth. However, in 2016–2018, the green total factor productivity of the Yangtze River Economic Zone fluctuated greatly. It shows that the regional economic development of the Yangtze River Economic Zone is restricted by resources and environmental factors to a certain extent, and has not yet formed a stable resource-environment friendly development model.
2. The spatial pattern of ecological efficiency in the upper, middle, and lower reaches of the Yangtze River Economic Zone is characterized by "ladder type". But the difference between the upper and middle reaches is small. During the sample period, the contribution of the technology gap rate in the middle reaches is higher, while that in the upper and lower reaches is increasing year by year. During this period, the contribution of the middle reaches is very different from that of the lower and upper reaches, which is the primary reason for the overall change of the technological gap rate of the Yangtze River Economic Zone.
3. Cities with higher green total factor productivity and lower green total factor productivity each form the characteristics of "club convergence" of spatial agglomeration. The spatial agglomeration effect of 11 provinces and cities in the Yangtze River Economic Zone can be roughly divided into three categories: the agglomeration effect of the upper reaches of the Yangtze River represented by 41 prefecture-level cities in Shanghai, Jiangsu, Anhui and Zhejiang provinces; The agglomeration effect of the middle reaches of the Yangtze River represented by 36 prefecture-level cities in Hubei, Hunan and Jiangxi provinces; the agglomeration effect of the lower reaches of the Yangtze River represented by 31 prefecture-level cities in Chongqing, Sichuan, Yunnan and Guizhou provinces. This shows that the geographical comparative advantage of industrial production in different regions determined by resource endowment conditions and economic development level is an important reason for the differences between provinces and cities.
4. Technological efficiency and technological progress efficiency have heterogeneous effects on different river basins in the upper, middle and lower reaches, and technological progress efficiency is conducive to promoting the evolution of green total factor productivity to a high level. Some areas in the upper reaches have paid less attention to technological efficiency, which has affected the development of green total factor productivity. Industrial green development is not a simple issue of technological efficiency, technological progress is also the proper meaning of promoting industrial green productivity growth.

According to the above empirical results, the following policy recommendations are put forward:

1. In order to solve the qualitative problem of green total factor productivity in the upper, middle and lower whirlpool cities of the Economic Zone and the phenomenon of "club convergence", the cities need to deepen the exchange and cooperation of ecological protection. At the same time, the Yangtze River Delta and other areas with high green total factor productivity should play a leading role in radiation, increase efforts to help cities with low state to build high-tech industries, and promote the ecological efficiency of surrounding areas. Industrial development planning and industrial policies in the middle, upper and lower reaches should meet the needs of realistic development, "urban governance, local policy", guided by superior industries and resources, and optimize the allocation of resources.
2. Encourage enterprises to improve their enthusiasm for technological research and development with policy benefits, and promote technological upgrading of traditional industries. Through the formulation of local medium and long-term talent training and talent introduction plans, the implementation of various welfare policies to attract high-tech talents to settle down. The ability of independent innovation of enterprises is still insufficient, and the efficiency of technological progress changes little, showing a slow growth trend. It shows that although the external economic environment is relatively bad, the government still adopts a relatively cumulative fiscal policy to optimize the efficiency of resource allocation and make the economic growth momentum.
3. In the long run, government regulation can only be used as an auxiliary means to maintain the stable development of the market. It is the inevitable choice for China’s economy to achieve sustainable and high-quality development by speeding up the promotion of enterprises’ independent innovation ability, improving the utilization efficiency of various factors of production such as labor force, land, capital and technology, and optimizing the industrial structure. On the premise of adhering to the concept of sustainable development, we should continue to maintain the better development of areas with high green total factor productivity, provide policy resources for promoting the rapid economic development of lower areas, and then realize the sustainable and coordinated development of a regional economy.
4. Play a vanguard and exemplary role in environmental protection, and establish and improve the coordinated protection mechanism of the Yangtze River ecological environment. We should actively change the mode of economic growth, change high input, high consumption, high pollution, low output, low quality and low efficiency into low input, low consumption, low pollution, high output, high quality and high efficiency, and change the extensive economic development model into intensive economic development model. We should change the "pure economic oriented" development model to the "sustainable development model of ecological civilization", actively promote regional exchanges and cooperation in industrial science and technology, ensure the effective promotion and diffusion of advanced technologies, so as to realize the industrial green elements in all regions.

## Supporting information

S1 Data (XLSX)

S1 File (XLSX)

S2 File (XLSX)

S3 File (XLSX)

S4 File (XLSX)
